# Wounds of Eye-Ball

**Published:** 1883-02

**Authors:** Peter A. Callan


ARTICLE IV.

### WOUNDS OF EYE-BALL.

BY PETER A. CALLAN, M. D.

CASE I. A. W., aged thirty-eight, private watchman, about one month ago while looking up toward the upper story of a house to see where some fallen glass had come from, was struck by a piece of window glass in the right eye. Patient came to the New York Eye and Ear Infirmary twenty-four hours after the accident. I found the following: right eye, incised wound one-half an inch in length beginning in cornea downward and outward and extending into sclerotic about an eighth of an inch; iris prolapsed at the limitus cornea, lens fully cataractous, reaction very slight. Put patient to bed and used ice-cold compresses for two days. At the expiration of that time put patient under ether and made an iridectomy at the wound, by enlarging along the line of the limitus, the original wound; removed enough iris tissue to prevent another prolapse; eye bandaged and patient kept on his back for three days. At the expiration of that time found wound healed; no reaction, no pain; patient discharged. He presents himself once a week, and the eye in no way impedes his working. At some future time shall operate for the removal of the cataract.

CASE II. Eva M. S., aged eleven, while running along the sidewalk two months ago with a milk pitcher in her hand, slipped and fell forward, breaking the pitcher and wounding her left eye. Patient was taken to one of our large hospitals and kept for over a month. When discharged she presented herself at the New York Eye and Ear Infirmary. Examination showed that the left upper lid had been incised by the broken pitcher, and on opening the lids and looking at the eye there were seen several prolapses of the iris extending over the upper half of the cornea and some at the upper limitus, with cicatrices in the region of the ciliary body. The eye was painful; lens cataractous and light perception not good. Under the circumstances enucleation of the eye-ball was advised to prevent further sympathetic inflammation of fellow eye. The parents were expecting it, inasmuch as a grandparent had lost an eye by injury and subsequently the fellow eye had become affected through sympathetic inflammation. Enucleation was performed, and patient now wears an artificial eye.

Case III. J. W., aged twenty-three, while getting off a street car, about the last week in November, slipped and fell, and one of the ribs of an umbrella which he was carrying entered the left orbital cavity through the lower lid, not directly touching the eye-ball as far as I could make out. Seven days after the injury he came to the infirmary with well-marked orbital cellulitis, the eye-ball being pressed down and outward. Cornea slightly hazy, considerable muco-purulent secretion from the conjunctival surfaces. Patient's general health was very poor. He objected to entering the infirmary just then, but promised to return in a few days. Returned in three days; the lids were, possibly, not so much swollen, but the secretion was worse and a well-marked ulcer appeared on the upper third of the cornea. Patient was put to bed and constant applications of boracic water, the daily use of a solution of nitrate of silver (five or ten grains, as the indications called for it), a solution of sulphate of atropia, gr. ij. three times daily to the eye, and a generous diet with alcoholic stimulants given to the patient. The entire cornea sloughed away, leaving the iris exposed. Patient began to suffer intense pain, which it took large doses of opium to partially alleviate. Pain so severe, and eye lost, it was decided to remove the eye, which my assistant, Dr. Agramonte, did in a very skillful manner. About the tenth day a secondary hemorrhage took place, which after much difficulty was stopped by liq. ferri. persulph. and compression. The orbital inflammation gradually subsided, and after about one month's treatment in the Infirmary he was discharged.

When seen soon after the injury all such patients should be put to bed, bowels opened, diet regulated, and to the affected eye, ice-cold lotions to prevent too great inflammation, which usually begins within twenty-four hours after the injury. The shock is sometimes very marked following an injury to the eye. If there is any foreign body to be seen in or near the wound it can be removed if not too great disturbance of the eye is necessary. A prolapsed iris may be replaced by the use of eserine locally and gentle manipulation. If this does not do it—and in the majority of cases it will not succeed—let it alone and at the expiration of three or four days it can be cut off and a bandage applied. We strongly object to the indiscriminate use of sulphate of atropia to an injured eye if the case is seen very soon after the accident. In case there is an incised wound of cornea with or without a prolapse the chances are that the lens capsule may be more or less torn, and some lens matter has become opaque from the aqueous humor penetrating through the torn capsule to the lens. When sulphate of atropia is used the lens is flattened and the change in shape disturbs the wound in capsule and permits more aqueous humor to reach the lens, rendering it more opaque; the lens swells and makes the case more complicated and serious. A surgeon assumes a great responsibility who has charge of a case where one eye has been lost through injury. If the foreign body—a piece of iron, brass, percussion-cap or any hard non-absorbable material—has penetrated into the eye and remains within, there is danger sooner or later that the fellow eye will become sympathetically affected. Or where an eye has been injured in such a manner that there is extensive laceration in and about the ciliary body, or in fact any portion of the eye-ball wounded in such a way that in healing the ciliary nerves are included in the cicatrix then there is danger to be feared of sympathetic inflammation of the fellow eye.

